# Identification of Telosma mosaic virus infection in *Passiflora edulis* and its impact on phytochemical contents

**DOI:** 10.1186/s12985-018-1084-6

**Published:** 2018-11-01

**Authors:** Shuangshuang Chen, Nannan Yu, Shaohuan Yang, Baoping Zhong, Hanhong Lan

**Affiliations:** School of Biological Sciences and Biotechnology, Minnan Normal University, Xianqianzhi street, Xiangcheng district, Zhangzhou, Fujian 363000 People’s Republic of China

**Keywords:** Telosma mosaic virus, *Passiflora edulis*, Infection, Phytochemical components, Antioxidative capacity

## Abstract

**Background:**

Viral disease has become the most severe constraint for the cultivation and production of *Passiflora edulis* in China*.* The infection of Telosma mosaic virus (TeMV), a potyvirus, and its effects on the phytochemical components of *P. edulis* remain largely unknown in China.

**Methods:**

*P. edulis* plants showing distorted leaves and severe mosaic skin on green fruit were identified with TeMV infection through traditional transmission electron microscopy, RT-PCR and modern small RNA sequencing (sRNA-seq) platform. The contents of phytochemical components and the activities of antioxidative enzymes were compared between virus-infected and virus-free *P. edulis* to confirm the effects of TeMV infection on host plant.

**Results:**

Firstly, approximately 700 nm linear virus particles, representing TeMV, were detected in infected *P. edulis* fruits and leaves with Electron microscopy. Partial coat protein genes of TeMV were successfully amplified by RT-PCR in infected *P. edulis* leaves and fruits but not in healthy plants. Abundant small interference RNAs (siRNAs) sequences, showing several characterizations, were specifically generated from the TeMV genome in infected plant fruits by sRNA-seq platform. Furthermore, fruit length, fruit thickness (wideness) and fruit weight decreased significantly due to TeMV infection. The levels of total protein and total sugar increased significantly; however, the level of total fat, total acid and vitamin C decreased obviously after TeMV infection. The level of total phenols, a secondary metabolite, was obviously higher in TeMV-infected than TeMV-free *P. edulis* fruit. The activities of superoxide dismutases (SOD) and catalases (CAT) obviously increased in TeMV-infected in comparison with healthy *P. edulis* fruit.

**Conclusions:**

TeMV infection adversely affected the development of *P. edulis* fruits, differently and selectively modulated the phytochemical components of *P. edulis* fruits. In turn, *P. edulis* plants enhanced their tolerance to the stress of TeMV infection by increasing the secondary metabolite level and the antioxidative capacity. This is of significant importance to understand the effects of TeMV infection on the biochemical changes and the antioxidant defense mechanism in *P. edulis*.

## Background

*Passiflora edulis*, an important *Passifloraceae* fruit, is cultivated throughout tropical and subtropical regions of the world, including China. It has been widely used in folk medicine to treat anxiety, insomnia, asthma, bronchitis and urinary infection. The constituents of different functional extracts include phenols, proteins, flavonoids, alkaloids, cyanogenic compounds, glycosides, vitamins, minerals and terpenoid compounds [1, 2]. Due to its medicinal properties to cure human of many health disorders and with high nutritional value, people have shown interest to include *P. edulis* in their diet and hence the demand for *P. edulis* has increased. On the other hand, the cultivation and production of *P. edulis* are severely affected by several diseases caused by fungal, bacterial and viral pathogen [3]. Among which, viral disease has been reported to cause most devastating effect for *P. edulis*. Viruses infecting *P. edulis* included members of the genus *Potyvirus*, *Cucumovirus*, *Begomovirus*, *Tymovirus*, *Cilevirus*, *Cilevirus*, *Carlavirus*, *Tobamovirus* and *Nepovirus* [4–12]. Telosma mosaic virus (TeMV), a potyvirus, was firstly reported to infect *Telosma cordata* plants in Vietnam, subsequently patchouli plants in Indonesia and recently passion fruit in Thailand, Haikou and Fujian of China [13–17]. TeMV-infected *P. edulis* showed severe symptoms, such as mosaic and distorted leaves, mosaic skin on green fruit and decreased fruit size. The effect of viruses infection on the chemical composition of host plants has been widely reported. *Indian cassava mosaic virus* (ICMV), *Plum pox virus* (PPV) and *Tomato leaf curl Palampur virus* (TLCPV) infection modified the composition of nutritive and bioactive compounds of plant host, *Momordica charantia*, *Prunus domestica* and *Cucurbita moschata*, respectively [15, 18, 19]. *Passion fruit woodiness virus* (PWV), another potyvirus, the most economically important viral disease of passion fruit plants in Brazil, can affect the content of phenolic compounds, an antioxidant molecule, in rinds [2]. However, up until now, there is no report about the effects TeMV infection on the phytochemical contents and the activities of antioxidative enzymes of *P. edulis* fruit.

The aims of the present study are (a) to identify the TeMV infection in *P. edulis* in Zhangzhou City of Fujian Province by traditional electron microscopy, RT-PCR and modern small RNA deep sequencing, (b) to examine the effect of TeMV infection on contents of phytochemicals in the fruits, (c) to investigate the role of individual antioxidative enzymes (SOD and CAT) in protection of *P. edulis* plants against oxidative damage caused by virus infection.

## Material and method

### RT-PCR

*P. edulis* samples (*n* = 50) showing severe mosaic and distorted leaves and mosaic skin on green fruit after 7 days were respectively collected from Zhangzhou (Xiangcheng district, Nanjing County, Zhaoan County and Pinghe County) and Longyan (Xinluo district and Wuping County) in Fujian. Total RNA was extracted from 0.1 g symptomatic leaves and fruits with Trizol reagent (Invitrogen, USA) following manufacturer’s instruction. The cDNA was prepared from total RNA using M-MLV reverse transcriptase and gene-specific primers (available upon request) in a final volume of 20 μL. Total RNA and primer were denatured at 70 °C for 10 min, then 10 μL of Master Mix (Beyotime, Shanghai, China) was added before incubating at 42 °C for 1 h, then at 70 °C for 10 min. The about 500-bp fragment for TeMV was amplified using 2 μL cDNA as the template in a final volume of 50 μL with InvitrogenTM SuperScriptTM IV VILOTM kit (ThermoFisher, USA) [20] with an initial denaturation at 94 °C for 3 min; followed by 35 cycles at 94 °C for 1 min, 56 °C for 30 s, 72 °C for 45 s and 72 °C for 10 min. Healthy *P. edulis* samples were used as a control.

### Electron microscopy

After confirmation by RT-PCR, infected and healthy *P. edulis* leaves and fruits were investigated for virus particles with the negative staining method as described by Brenner with moderate adjustment [21]. Virus particle was observed under H-7650 Hitachi transmission electron microscope at 80 kV.

### Small RNA sequencing and analysis

Library construction and sequencing of small RNA extracted from infected and healthy *P. edulis* fruits (*n* = 3) were performed by Novogene technology co. LTD (Beijing, China) as described previously [22, 23]. Briefly, following PAGE purification of small RNA molecules and adaptor ligation to their 5′ and 3′ ends, small RNA molecules were amplified using the adaptor primers, and fragments approximately 90 bp were isolated from the agarose gel. The purified DNA was utilized directly for small RNA sequencing analysis using Illumina’s Solexa Sequencer. Raw data sets for the small RNA were analyzed. In brief, adaptor sequences were trimmed, and small RNA reads without an identifiable linker were removed. The remaining reads were filtered by length and reads > 32 nt or < 18 nt were discarded. To identify siRNAs derived from TeMV, we aligned all the cleaned reads with the software Bowtie v.0.12.7 with a parameter of 0 mismatch to the viral genome sequences. The downstream analyses of reads aligned with viral genome were performed using Perl scripts and Excel. The average depth was calculated as the total number of nucleotides of the aligned reads divided by the read-covered positions on the reference genome. The genome coverage represented the proportion of read-covered positions against the genome length. Single-base resolution maps along with viral genome were created using Bowtie tools and in-house Perl scripts. siRNAs were assembled de novo into long contigs using Velvet software with a *k-mer* of 17 or 19. Assembled contigs were used to search the GenBank/EMBL/DDBJ database using BLASTn or BLASTx. Coverage and distribution of virus specific contigs by siRNAs were determined using the program MAQ under default parameters.

### Phytochemical analysis

Nine samples (*n* = 9) positive for the presence of TeMV infection were uses for phytocheical analysis. Fruit juice obtained from healthy and infected fruit respectively was homogenized with a homogenizer for phytochemical analysis. To determine of total protein, 100 g fruit juice were digested with 5.0 ml of 2 M NaOH at 100 °C for 60 min. After centrifugation at 160000 g for 10 min, the protein content in the supernatant was detected with the method of the Folin-Phenol reagent using bovine serum albumin (BSA, Sigma, USA) as a standard [24]. To detect total sugar, 100 g fruit juice was centrifuged at 5000 rpm for 30 min. The supernatant was taken for the estimation of total sugar level with the Phenol–Sulfuric acid method using glucose as a standard [25], a most reliable method among all the quantitative assays for carbohydrate estimation. As for total fat detection, 100 g fruit juice was evaporated to dryness with a dish in boiling water, then moved into a filtration paper cylinder and extracted with petroleum ether with a backflow of 6 times/hour with a Soxhlet extractor in boiling water [26]. As for total acid detection, 100 g fruit juice was boiled in boiling water for 30 min, diluted with water to 250 ml, then was filtered with filter paper. The filtrate was used for total acid test by acid-base titration with phenothalin solution used as an indicator for end-point pH [27]. Vitamin C in the sample was determined by titrating its aqueous extract with a solution of 2,6-dichlorophenol-indophenol dye to a faint pink endpoint [28]. The total phenolic content of the samples was determined with the Folin-Ciocalteus reagent method with gallic acid as a standard substance [29]. After extraction of 100 g fruit juice with ethanolic solution, 1.0 ml of extracts and 1.0 ml of diluted Folin-Ciocalteu reagent were mixed. After 3 min, 1.0 ml of 10% sodium carbonate was added to the mixture and was allowed to stand for 1 h at 25 °C. The absorption was measured at 765 nm using UV spectrophotometer. The total phenolic content was expressed as milligrams of gallic acid equivalent per gram of fruit juice (mg/g).

### Superoxide dismutase (SOD) and catalase (CAT) assays

The SOD activity of healthy and infected fruit juice (*n* = 9) was assayed as described by Misra with moderate modification [30]. In brief, 100 mg fruit juice was homogenized in 100 mM K-phosphate buffer (pH 7.8). The extract was centrifuged at 22000 g for 10 min at 4 °C. The supernatant was dialyzed in cellophane membrane tubes with cold extraction buffer for 4 h, then, used for the assay. The assay mixture in a total volume of 3 ml contained 50 mM sodium carbonate/bicarbonate buffer (pH 9.8), 0.1 mM EDTA, 0.6 mM epinephrine and enzyme. Epinephrine was the last component to be added. The adrenochrome formation was recorded at 475 nm with a T9CS spectrophotometer (Beijing). One unit of SOD activity was expressed as the amount of enzyme required to cause 50% inhibition of epinephrine oxidation under the experimental conditions.

Also, the CAT activity of fruit juice obtained above was assayed as described by Beers with moderate modification [31]. In brief, 100 mg fruit juice was homogenized in 5 ml of 50 mM Tris/NaOH buffer (pH 8.0). The homogenate was centrifuged at 22000 g for 10 min at 4 °C; followed by dialysis, the supernatant was used for enzyme assay. The decomposition of H_2_O_2_ was followed at 240 nm by observing decrease in absorbance. Enzyme specific activity is expressed as μmol of H_2_O_2_ oxidized min^− 1^ (mg protein)^− 1^.

### Statistical analysis

All data were expressed as mean and standard deviation (σ) of at least three replicate samples. The data were analyzed using one-way analysis of variance (ANOVA). Significant differences were calculated at *p* < 0.05 using least significant difference (LSD) test with SPSS statistics software 17.0.

## Results

### TeMV infection in showing symptoms *P. edulis*

**Fig. 1:**
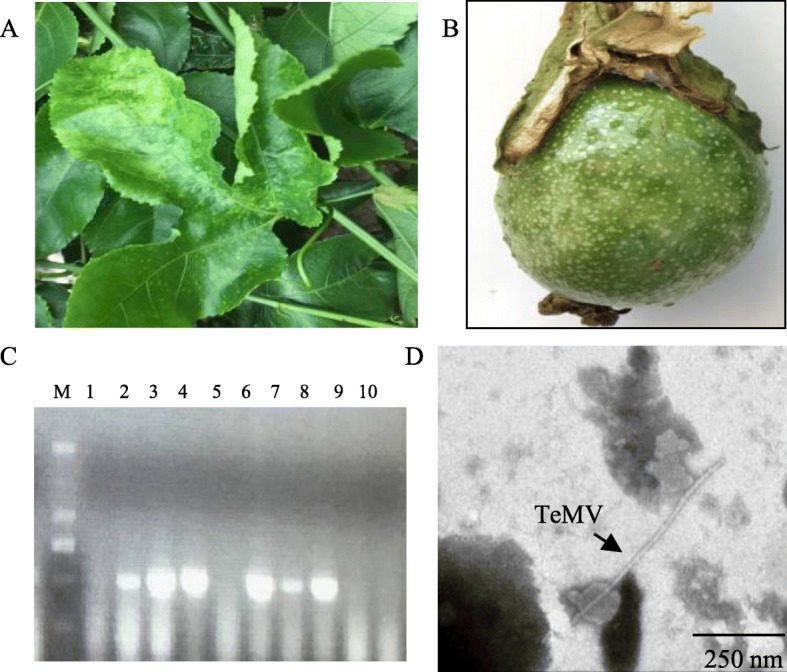
Confirmation of *P. edulis* leaves and fruits infected with TeMV by RT-PCR and electron microscopy. **a** and **b**, Symptoms of *P. edulis* infected with TeMV. The infected plants showing severe mosaic and distortion leaves (**a**) and mosaic skin and granule preiectionon green fruit (**b**). **c**, Agarose gel electrophoresis of RT-PCR products of partial TeMV CP gene. M, DNA mareker D2000; 1–10, RT-PCR products of TeMV CP gene from infected leaves and fruits, respectively. **d**, Electron microscopy of *P. edulis* fruits infected with TeMV showing virus particle morphology through the negative staining. Bars, 250 nm

Total 300 *P. edulis* samples (*n* = 300) showing severe mosaic and distorted leaves and mosaic skin on green fruit (Fig. 1a and b) caused by viral infection after 7 days were collected from Zhangzhou (Xiangcheng district, Nanjing County, Zhaoan County and Pinghe County) and Longyan (Xinluo district and Wuping County) in Fujian. TeMV infection was confirmed by RT-PCR and by H-7650 Hitachi transmission electron microscopy. Our results showed that partial coat protein genes of TeMV were successfully amplified by RT-PCR in average 8.7% (26/300) leaves and fruit of *P. edulis* showing symptoms but not in healthy plants from Longyan and Zhangzhou cities (Table 1, Fig. 1c). Furthermore, electron microscopy suggested that approximately 700 nm linear TeMV particles existed in viruses-infected fruit (Fig. 1d) and leaves (data not shown) of *P. edulis* plants.

**Table 1:** Infection rates of *P. edulis* fruit with TeMV as detected by RT-PCR at different regions in Fujian (*n* = 300)

City	District or County	Infection rates (%)
Zhangzhou	Xiangcheng	14
	Nanjing	6
	Zhaoan	10
	Pinghe	8
Longyan	Xinluo	8
	Wuping	6

**Fig. 2:**
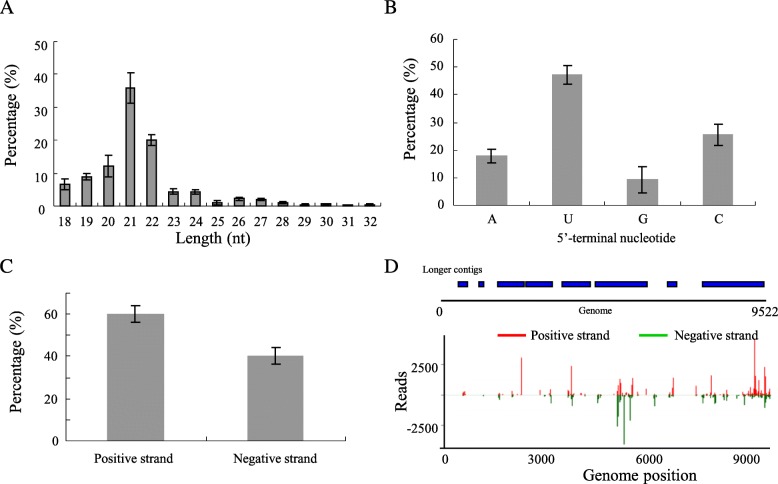
Characterizations of siRNAs (18–32 nt) derived from TeMV in *P. edulis* fruits through small RNA deep sequencing platform. **a**, Size distribution of siRNAs matching to TeMV in infected *P. edulis* fruits. **b,** Nucleotide bias of 5′-terminal nucleotide of siRNAs matching to TeMV in infected *P. edulis* fruits. **c**, Polarity distribution of siRNAs matching to TeMV in infected *P. edulis* fruits. “+” and “-” indicate siRNAs derived from positive and negative genomic strands, respectively. **d,** Coverage (up panel) and profile (down panel) of siRNAs along the reference TeMV genome in infected *P. edulis* fruits. **a, b, c** and **d**; values are mean of three independent experiments

Infection of viral pathogens in plants was characterized by the generation of small interference RNA derived specifically from viral genome (vsiRNAs). Thus, sRNA-seq was used to investigate the production of siRNAs in *P. edulis* infected with TeMV after 7 days. We aligned small RNA to the TeMV genome sequence using the software Bowtie v.0.12.7 with a parameter of 0 mismatch. In total, 11,417,548 small RNA sequences were generated, and siRNAs specifically derived from TeMV comprised 0.44% of the total small RNAs from viral infected *P. edulis.* TeMV siRNAs was rarely identified in healthy *P. edulis* fruit (data not shown)*.* Furthermore, TeMV siRNAs were predominantly 21-nt and 22-nt long (Fig. 2a). TeMV siRNAs demonstrated a clear tendency to begin with uracil (U), adenine (C), as compared with cytosine (A) and guanidine (G) (Fig. 2b). TeMV siRNAs were produced nearly equally from the positive and the negative strands of viral genomes (Fig. 2c). TeMV siRNAs had a continuous but heterogeneous (Hot spot and Cold spot) distribution along the genomes (Fig. 2d, down panel). Furthermore, the 8 longer contigs assembled with Velvet software with a *k-mer* of 17 or 19 covered 73.2% of the reference genome with an average depth 9.5 (Fig. 2d, up panel). Taken together, our results demonstrated that *P. edulis* showing mosaic and distorted leaves and mosaic skin on green fruit indeed was infected by TeMV.

### The effects of TeMV infection on the physical properties of *P. edulis*

To confirm the effects of TeMV infection on the development of *P. edulis,* physical properties (fruit length, fruit thickness and fruit weight) of *P. edulis* (*n* = 9) were evaluated in TeMV-infected and TeMV-free plants fruits. Our results revealed that fruit length, fruit thickness (wideness) and fruit weight decreased significantly in virus-infected *P. edulis* plants when compared with that of virus-free *P. edulis* plants (Table 2). The fruit length decreased from 6.14 cm to 3.74 cm; the fruit thickness decreased from 4.82 cm to 2.93 cm and the fruit weight decreased from 50.98 g to 31.02 g (Table 2). The productivity per hectare of *P. edulis* is about 15,000 kg, thus, the losses of the productivity per hectare of *P. edulis* caused by TeMV infection was estimated to 470 kg. Token together, TeMV infection affected adversely the development and the productivity of *P. edulis* fruits.

**Table 2:** The effects of TeMV infection on the physical properties of *P. edulis* fruit (Mean ± σ, *n* = 9)

	Fruit length (cm)	Fruit thickness (cm)	Fruit weight (g)
TeMV-infected	3.74 ± 0.60	2.93 ± 0.64	31.02 ± 5.10
TeMV-free	6.14 ± 0.71	4.82 ± 0.65	50.98 ± 4.02
*p*-value	< 0.01	< 0.01	< 0.01

### The effects of TeMV infection on the phytochemical components of *P. edulis*

**Fig. 3:**
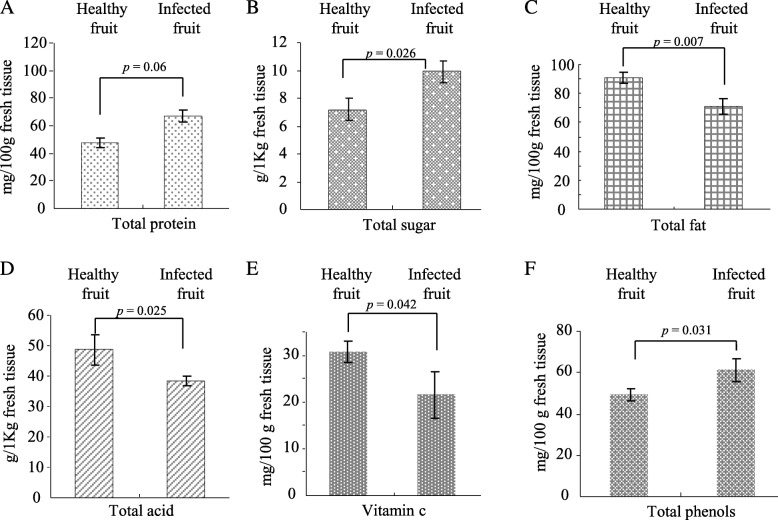
The effects of TeMV infection on the phytochemical components of *P. edulis* fruits. Total protein (**a**), total sugar (**b**), total fat (**c**); total acid (**d**); Vitamin C (**e**) and total phenols (**f**) were detected from health and infected *P. edulis* fruits. Values are mean ± standard deviation (σ) of nine independent experiments

A variety of adverse environmental conditions or stresses including the pathogen were known to cause the changes of phytochemical components to host plants either. Several phytochemical components of virus-infected and virus-free *P. edulis* fruits juices were analyzed and compared. Analysis of three main phytochemical energy substances revealed that the total protein and sugar contents increased by 39.6% and 19.1% respectively; however, the total fat contents decreased by 21.6% in virus-infected *P. edulis* fruits compared with that of virus-free *P. edulis* fruits (Fig. 3a, b and c). Furthermore, the total acid and vitamin C contents were lower in the virus-infected *P. edulis* fruits as compared with healthy *P. edulis* fruits (Fig. 3d and e). Phenolic compounds are some among the most influential and widely distributed secondary products in plants. Our results showed that the total phenol contents were significantly higher in the virus-infected *P. edulis* fruits, with 19.1% increase over the healthy *P. edulis* fruits (Fig. 3f). Thus, the phytochemical components were differently and selectively modulated by the infection of TeMV in *P. edulis* fruits.

### The effects of TeMV infection on the activities of antioxidative enzymes of *P. edulis*

**Fig. 4:**
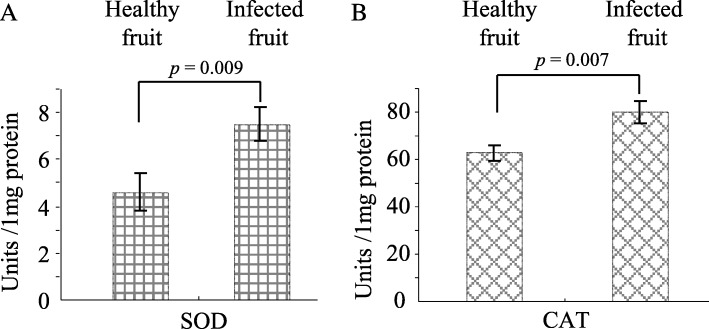
The effects of TeMV infection on the activities of antioxidative enzymes of *P. edulis* fruits. SOD (**a**) and CAT (**b**) were detected from health and infected *P. edulis* fruits. Values are mean ± standard deviation (σ) of nine independent experiments

SOD is the key antioxidative enzyme and catalyzes dismutation of superoxide free radical (O_2_^−^) into H_2_O_2_ and O_2_. In turn, CAT break down H_2_O_2_ in the living. Thus, the activities of antioxidative enzymes (SOD and CAT) were analyzed in virus-infected and virus-free *P. edulis* fruits. Our results demonstrated that there was an obviously increase in the activities of SOD by 63.0% and 27.3% increase in CAT (Fig. 4). Therefore, these results imply that to enhance their tolerance to stresses of TeMV infection, as a feedback mechanism, *P. edulis* plant increases its antioxidative capacity.

## Discussion

Potyvirus is the largest genus of plant viruses causing significant losses in a wide range of crops in tropical and subtropical regions of the world [32]. Growing evidences showed that diverse potyvirus species could infect *P. edulis* plants in many parts of the world [33]. Telosma mosaic virus (TeMV), a potyvirus, was reported to infect *Telosma cordata* plants in Vietnam, subsequently patchouli plants in Indonesia and recently passion fruit in Thailand and Haikou and Fujian province of China [13–17]. However, up until now, the infection of TeMV and its effects on phytochemical contents of *P. edulis* plant especially fruits remain largely unknown.

The identification of a virus typically requires the application of a number of methods including physical, biological, serological and molecular methods. In this present study, firstly, TeMV infection associated with mosaic and distorted leaves and mosaic skin on green fruit (Fig. 1a and b) was identified with traditional electron microscope and polymerase chain reaction (PCR) (Fig. 1c and d, Table 1). However, the two detection methods depend on prior knowledge of morphological characteristics of virus particles or specific sequence of the potential virus. Recently, next generation high-throughput parallel sequencing platforms have proved to be highly efficient in identification of diverse plant and animal viruses [34–39]. Thus, sRNA-seq was used to identify TeMV infection in *P. edulis* plants. We analyzed and characterized some common features of the virus-derived small interfering RNA (vsiRNAs) specifically from TeMV, including the amount (0.44%), the length distribution (mainly 21-nt and 22-nt), the bias of first nucleotide (mainly G and C), the polarity distribution (equally from positive and negative strands) and frequency distribution (hot and cold spots) of vsiRNAs along the TeMV genome, the coverage (73.2%) and the average depth (9.5) (Fig. 2). Taken together, these methods mentioned above confirmed the infection of TeMV in *P. edulis* plants.

TeMV infection adversely affected the developments of *P. edulis* fruits (Table 2). However, the effects of viral infection on the phytochemical components remain unknown. The total proteins and total sugars levels increased significantly in TeMV-infected *P. edulis* fruit compared with TeMV-free *P. edulis* fruit (Fig. 3a and b). Similar observations were also reported for begomovirus-infected pumpkin/bitter gourd and for potyvirus-infected plum, where increase in the level of total proteins and total sugars was caused by virus infection, respectively [18, 19, 40]. Host nutrition can play a key role for the outcome of pathogen infections in host, since it is critical for immune-defense and resistance to pathogens. Poor nutrition, in particular protein or sugar depletion, is a major factor in high incidence and host mortality due to infectious diseases. Thus, the increased total proteins and total sugars may be implicated in pathogen defense. In contrast, total fat and total acid was decreased due to TeMV infection (Fig. 3c and d). Thus, virus infection could differently and selectively modulate the nutrition components (three primary metabolites) of *P. edulis* fruit. Recently, more evidence showed that changes of secondary metabolites were involved in host plant resistance in response to invading pathogens. Phenols, a secondary metabolite, played important roles in host-pathogen interaction, disease development and defence reaction of infected plants [41]. Our results showed that the level of total phenols was obviously higher in TeMV-infected than TeMV-free *P. edulis* fruit (Fig. 3f). Therefore, the increased quantity of total phenols in virus-infected *P. edulis* fruit presumably appears to contribute towards the resistance against viral infection. Virus pathogens are known to cause oxidative damage such as tissue necrosis to plants by triggering excess production of reactive oxygen species (ROS), which in turn could defend against invading pathogens at moderate level [42, 43]. Plant cells are protected against the oxidative damage caused by ROS through a complex antioxidant system, comprising antioxidants like ascorbic acid (Vitamin C, Vc) and antioxidant enzymes like superoxide dismutases (SOD) and catalases (CAT).Very few reports are available for antioxidative enzymes activity in plants subjected to biotic stresses especially, viral infection. SOD is the key antioxidative enzyme and catalyzes dismutation of superoxide free radical (O_2_^−^) into H_2_O_2_ and O_2_. In turn, CAT break down H_2_O_2_ in the living. In this study, a significant decrease of Vc (30%) (Fig. 3e) but an obvious increase of SOD (63%) and CAT (27%) activities were observed in the virus-infected *P. edulis* fruit (Fig. 4). Our observations are in agreement with report for geminivirus-infected bitter gourd and tomato leaf curl palampur virus-infected pumpkin [18, 40].

## Conclusion

The studies presented here confirmed the infection of TeMV in *P. edulis* plant in Zhangzhou City of Fujian Province in China at molecular level through traditional and modern bio-technologies and for the first time assessed its impacts on phytochemicals components and antioxidative enzymes activities of diseased plants. This is of significant importance to understand the effects of TeMV infection on the biochemical changes and the antioxidant defense mechanism in plants after virus infection.

## Abbreviations

CAT: catalases
PWV: Passion fruit woodiness virus
siRNAs: small interference RNAs
SOD: superoxide dismutases
sRNA-seq: small RNA sequencing
sRNA-seq: small RNA sequencing
Vc: vitamin c
TeMV: Telosma mosaic virus
vsiRNAs: small interference RNA derived specifically from viral genome

## References

[1] Zibadi S, Watson RR (2004). Passion fruit (*Passiflora edulis*). Evidence-Based Integrative Medicine.

[2] Zeraik ML, Serteyn D, Deby-Dupont G, Wauters JN, Tits M, Yariwake JH (2011). Evaluation of the antioxidant activity of passion fruit (*Passiflora edulis* and *Passiflora alata*) extracts on stimulated neutrophils and myeloperoxidase activity assays. Food Chem.

[3] Fischer IH, Rezende JA (2008). Diseases of passion flower (*Passiflora* spp.). Pest Technol.

[4] Fontenele RS, Abreu RA, Lamas NS, Alves-Freitas DM, Vidal AH, Poppiel RR (2018). Passion fruit chlorotic mottle virus: molecular characterization of a new divergent geminivirus in Brazil. Viruses.

[5] Polston JE, Londoño MA, Cohen AL, Padilla-Rodriguez M, Rosario K, Breitbart M (2017). Genome sequence of *Euphorbia mosaic virus* from passionfruit and *Euphorbia heterophylla* in Florida. Genome announcements.

[6] Vaca-Vaca JC, Carrasco-Lozano EC, López-López K (2017). Molecular identification of a new begomovirus infecting yellow passion fruit (*Passiflora edulis*) in Colombia. Arch Virol.

[7] Fukumoto T, Nakamura M, Rikitake M, Iwai H (2012). Molecular characterization and specific detection of two genetically distinguishable strains of *East asian passiflora virus* (EAPV) and their distribution in southern Japan. Virus Genes.

[8] Coutts BA, Kehoe MA, Webster CG, Wylie SJ, Jones RA (2011). Indigenous and introduced potyviruses of legumes and *Passiflora* spp. from Australia: biological properties and comparison of coat protein nucleotide sequences. Arch Virol.

[9] Song YS, Ryu KH (2011). The complete genome sequence and genome structure of passion fruit mosaic virus. Arch Virol.

[10] Parrella G, Lanave C (2009). Identification of a new pathotype of *Bean yellow mosaic virus* (BYMV) infecting blue passion flower and some evolutionary characteristics of BYMV. Arch Virol.

[11] Spiegel S, Zeidan M, Sobolev I, Beckelman Y, Holdengreber V, Tam Y (2007). The complete nucleotide sequence of Passiflora latent virus and its phylogenetic relationship to other carlaviruses. Arch Virol.

[12] Nascimento AVS, Santana EN, Braz ASK, Alfenas PF, Pio-Ribeiro G, Andrade GP (2006). Cowpea aphid-borne mosaic virus (CABMV) is widespread in passionfruit in Brazil and causes passionfruit woodiness disease. Arch Virol.

[13] Ha C, Coombs S, Revill PA, Harding RM, Vu M, Dale JL (2008). Design and application of two novel degenerate primer pairs for the detection and complete genomic characterization of potyviruses. Arch Virol.

[14] Noveriza R, Suastika G, Hidayat SH, Kartosuwondo U (2012). *Potyvirus* associated with mosaic disease on patchouli (*Pogostemon cablin* (Blanco) Benth.) plants in Indonesia. J ISSAAS.

[15] Chiemsombat P, Prammanee S, Pipattanawong N (2014). Occurrence of *Telosma mosaic virus* causing passion fruit severe mosaic disease in Thailand and immunostrip test for rapid virus detection. Crop Prot.

[16] Yang K, Yan H, Song L, Jin P, Miao W, Cui H. Analysis of the complete genome sequence of a potyvirus from passion fruit suggests its taxonomic classification as a member of a new species. Arch Virol. 2018:1–4. 10.1007/s00705-018-3885-8 29789942

[17] lixue X, xiaoyan Z, shan Z, lijie Z, tao L (2017). Molecular identification and specific detection of *Telosma mosaic virus* infecting passion fruit. Sci Agric Sin.

[18] Jaiswal N, Singh M, Dubey RS, Venkataramanappa V, Datta D (2013). Phytochemicals and antioxidative enzymes defence mechanism on occurrence of yellow vein mosaic disease of pumpkin (*Cucurbita moschata*). 3 Biotech.

[19] Usenik V, Kastelec D, Stampar F, Virscek Marn M (2014). Effect of *Plum pox virus* on chemical composition and fruit quality of plum. J Agr Food Chem.

[20] Lan H, Hong X, Huang R, Lin X, Li Q, Li K (2018). RNA interference-mediated knockdown and virus-induced suppression of troponin C gene adversely affect the behavior or fitness of the green rice leafhopper, *Nephotettix cincticeps*. Arch Insect Biochem Physiol.

[21] Brenner S, Horne RW (1959). A negative staining method for high resolution electron microscopy of viruses. Biochim Biophys Acta.

[22] Lan H, Chen H, Liu Y, Jiang C, Mao Q, Jia D (2016). Small interfering RNA pathway modulates initial viral infection in midgut epithelium of insect after ingestion of virus. J Virol.

[23] Lan H, Wang H, Chen Q, Chen H, Jia D, Mao Q (2016). Small interfering RNA pathway modulates persistent infection of a plant virus in its insect vector. Sci Rep.

[24] Lowry OH, Rosebrough NJ, Farr AL (1951). Protein measurement with the Folin phenol reagent. J Biol Chem.

[25] Jain VardhamanMulchand, Karibasappa GundabakthaNagappa, Dodamani ArunSuresh, Mali GauraoVasant (2017). Estimating the carbohydrate content of various forms of tobacco by phenol-sulfuric acid method. Journal of Education and Health Promotion.

[26] Zhang CW, Wang CZ, Tao R (2016). Analysis on the physicochemical properties of ginkgo biloba leaves after enzymolysis based ultrasound extraction and soxhlet extraction. Molecules.

[27] Patnaik P (2004). Dean’s Anal Chem Handbook, 2nd ed..

[28] AOAC (1984). Official methods of analysis 14th ed.

[29] Sir Elkhatim KA, Elagib RA, Hassan AB. Content of phenolic compounds and vitamin C and antioxidant activity in wasted parts of Sudanese citrus fruits. Food Sci Nutr. 2018. 10.1002/fsn3.660 PMC6060895 30065822

[30] Misra HP, Fridovich I (1972). The role of superoxide anion in the autoxidation of epinephrine and a simple assay for superoxide dismutase. J Biol Chem.

[31] Beers RF, Sizer IW (1952). Colorimetric method for estimation of catalase. J Biol Chem.

[32] Ivanov KI, Eskelin K, Lõhmus A, Mäkinen K (2014). Molecular and cellular mechanisms underlying potyvirus infection. J Gen Virol.

[33] Taylor RH, Greber RS. Passion fruit woodiness virus. Description of plant virus no.122. In: Descriptions of plant viruses. Commonw. Mycol. Inst./Assoc. Appl. Biol., Kew, Surrey, England. 1973.

[34] Kreuze JF, Perez A, Untiveros M, Quispe D, Fuentes S, Barker I (2009). Complete viral genome sequence and discovery of novel viruses by deep sequencing of small RNAs: a generic method for diagnosis, discovery and sequencing of viruses. Virology.

[35] Li R, Gao S, Hernandez AG, Wechter WP, Fei Z, Ling KS (2012). Deep sequencing of small RNAs in tomato for virus and viroid identification and strain differentiation. PLoS One.

[36] Zheng Y, Gao S, Padmanabhan C, Li R, Galvez M, Gutierrez D (2017). VirusDetect: an automated pipeline for efficient virus discovery using deep sequencing of small RNAs. Virology.

[37] Wang F, Sun Y, Ruan J, Chen R, Chen X, Chen C, et al. Using small RNA deep sequencing data to detect human viruses. Biomed Res Int. 2016;2016. 10.1155/2016/2596782 PMC4811048 27066498

[38] Niu X, Sun Y, Chen Z, Li R, Padmanabhan C, Ruan J (2017). Using small RNA-seq data to detect siRNA duplexes induced by plant virus. Genes.

[39] Wu Q, Luo Y, Lu R, Lau N, Lai EC, Li WX (2010). Virus discovery by deep sequencing and assembly of virus-derived small silencing RNAs. PNAS.

[40] Raj SK, Khan MS, Singh R, Kumari N, Prakash D (2005). Occurrence of yellow mosaic geminiviral disease on bitter gourd (*Momordica charantia*) and its impact on phytochemical contents. Int J Food Sci Nutr.

[41] Treutter D (2006). Significance of flavonoids in plant resistance: a review. Environ Chem Lett.

[42] Wu J, Yang R, Yang Z, Yao S, Zhao S, Wang Y (2017). ROS accumulation and antiviral defence control by microRNA528 in rice. Nature plants.

[43] Baker CJ, Orlandi EW (1995). Active oxygen in plant pathogenesis. Annu Rev Phytopathol.

